# Scent of a killer: How could killer yeast boost its dispersal?

**DOI:** 10.1002/ece3.7534

**Published:** 2021-05-01

**Authors:** Claudia C. Buser, Jukka Jokela, Oliver Y. Martin

**Affiliations:** ^1^ Institute of Integrative Biology ETH Zürich Zürich Switzerland; ^2^ Department of Aquatic Ecology Eawag Dübendorf Switzerland; ^3^ Department of Biology ETH Zürich Zürich Switzerland

**Keywords:** attraction, dispersal, *Drosophila*, dsRNA virus, killer yeast

## Abstract

Vector‐borne parasites often manipulate hosts to attract uninfected vectors. For example, parasites causing malaria alter host odor to attract mosquitoes. Here, we discuss the ecology and evolution of fruit‐colonizing yeast in a tripartite symbiosis—the so‐called “killer yeast” system. “Killer yeast” consists of *Saccharomyces cerevisiae* yeast hosting two double‐stranded RNA viruses (M satellite dsRNAs, L‐A dsRNA helper virus). When both dsRNA viruses occur in a yeast cell, the yeast converts to lethal toxin‑producing “killer yeast” phenotype that kills uninfected yeasts. Yeasts on ephemeral fruits attract insect vectors to colonize new habitats. As the viruses have no extracellular stage, they depend on the same insect vectors as yeast for their dispersal. Viruses also benefit from yeast dispersal as this promotes yeast to reproduce sexually, which is how viruses can transmit to uninfected yeast strains. We tested whether insect vectors are more attracted to killer yeasts than to non‑killer yeasts. In our field experiment, we found that killer yeasts were more attractive to *Drosophila* than non‐killer yeasts. This suggests that vectors foraging on yeast are more likely to transmit yeast with a killer phenotype, allowing the viruses to colonize those uninfected yeast strains that engage in sexual reproduction with the killer yeast. Beyond insights into the basic ecology of the killer yeast system, our results suggest that viruses could increase transmission success by manipulating the insect vectors of their host.

## Graphical abstract

Some yeasts are more attractive to *Drosophila* than others. Because in other systems vector‐borne parasites can influence vector attraction, we consider whether certain viruses harbored by yeasts could affect observed variation. Considering our results—yeast infected with viruses attracts more *Drosophila* than uninfected yeasts—we discuss whether this observed variation is due to manipulation. In addition to olfactory cues used to locate yeast food sources, *Drosophila* vectors might also be influenced by the virus infections present within yeasts.

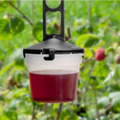

## 1 INTRODUCTION

Non‐motile microorganisms, such as the yeast *Saccharomyces cerevisiae*, actively attract vectors to disperse between spent and fresh ephemeral fruits. Interaction between common yeasts and the fruit flies has been used as an example of niche construction and can be beneficial for both species involved (Buser et al., 2014; Christiaens et al., 2014). Yeast attracts *Drosophila* flies to volatile compounds that it produces dispersing with the flies to new fruits (Becher et al., 2012; Begon, 1982; Buser et al., 2014). Some *S. cerevisiae* strains are known to be more attractive to *Drosophila* than others (Buser et al., 2014). Although mechanisms behind this variation remain unknown (Gunther & Goddard 2019), attractiveness does not seem to be linked to phylogenetic relatedness as both attractive and repulsive yeasts are found in different clades (Arguello et al., 2013; Becher et al., 2018; Buser et al., 2014; Gayevskiy et al., 2016; Peter et al., 2018).

Viruses are typically viewed as pathogens, but beneficial virus–host interactions have been described in many insects, plants, bacteria, and fungi (reviewed in Roossinck, 2011). Two *S. cerevisiae* viruses, the M satellite dsRNAs and the corresponding L‐A dsRNA helper virus, are seen as conditional mutualists to its host, as in combination they turn the infected yeast cells into lethal toxin‐producing “killer yeast” (Roossinck, 2011; Wickner, 1996). It is the M satellite dsRNA coding for a single protein that is responsible for toxin production (Zhu et al., 1993). The synthesized toxins are lethal to other yeast strains and thus provide a competitive advantage to the virus‐hosting “killer” strain. Crucially, the satellite virus renders the “killer” strain immune to the toxin that is produced in the cell. In this context, Boynton (2019) asked what additional benefits there might be for yeasts of hosting killer toxin‐producing viruses beyond interference competition. We suggest that an additional benefit might be that these viruses promote yeast dispersal by attracting more vectors to killer yeast infected fruits.

In nature, no evidence for extracellular transmission of dsRNA viruses infecting yeasts has been found. Therefore, these viruses strongly depend on the well‐being of the yeasts. Non‐motile yeasts need to disperse to leave spent and colonize new ephemeral fruits and thus enhance their reproductive success. Virus dispersal success thus depends completely on the success of the yeast in attracting insect vectors. In order to increase dispersal to new habitats, both viruses therefore could benefit if the yeast host is more attractive to vectors.

Dispersal also helps viruses to colonize new yeast strains. Viruses infect new host genotypes when germinated yeast spores fuse. Although *S. cerevisiae* has a strong tendency to inbreed (Goddard et al., 2010), an increased probability to outbreed (Reuter et al., 2007), spore release (Coluccio et al., 2008), and interstrain mating (Stefanini et al., 2016) seems to be promoted in insect intestines.

Parasites have been found to increase transmission and spread by altering host behavior in a broad variety of systems (Moore, 2002; Thomas et al., 2010). There are many different ways parasites are reported to manipulate host phenotype to increase transmission (Holmes & Bethel, 1972; reviewed in Hurd, 2003; Koella et al., 1998, Lefevre & Thomas, 2008; Thomas et al., 2002). These can include manipulation of their present host to be more attractive to prospective vectors (Busula et al., 2017; Cornet et al., 2013; De Moraes et al., 2014). As viruses have limited mobility, many depend on vectors for their dispersal and/or transmission. Most examples are found in plant‐virus systems where insects function as vectors (Whitfield et al., 2015). For example, cucumber mosaic virus attracts aphid vectors by inducing higher volatile release by the host plant (Mauck et al., 2010).

Here, we propose a novel hypothesis for this specific tripartite symbiosis. We suggest that viruses could manipulate attractiveness of killer yeasts to vectors to increase their own transmission to new hosts. In addition to verbal arguments, we use a field experiment to investigate general attractiveness of killer and non‐killer yeast to *Drosophila* vectors. We then discuss our observations as a starting point for further studies on whether enhanced attraction is due to virus manipulation.

## 2 MATERIALS AND METHODS

We tested the attraction of six *S. cerevisiae* strains with and without killer phenotype toward Drosophilidae. We used three distinct killer yeast strains and three different non‐killer strains of *S. cerevisiae* (killer yeast strains: YJM4541b (K1), CLIB294_1b (K1), Y12_1b (K28); non‐killer yeast strains: I14_1b, UC1_1b, NCYC_2743, Liti et al., 2009; Peter et al., 2018; Pieczynska et al., 2013, Table S1) each replicated six times. We inoculated 10^4^ yeast cells of each strain into 50 ml grape juice (homogenized and autoclaved Urpress Weiss from Rimuss). After 24 hr of inoculation at 28°C, we distributed the fermenting juice samples to *Drosophila* traps (Drosal^®^ Pro, Andermatt Biogarten) and randomly placed the 36 traps in a vineyard (Schipf: 47.291925, 8.601796; see Figure S1). Three traps of plain grape juice served as controls. After 72 hr, we collected the 39 traps and counted the total number of Drosophilidae and determined the species and sex of trapped flies.

### 2.1 Statistical analysis

A total of 6,361 insects were caught in the traps. Almost all (*n* = 6,315) belonged to the family Drosophilidae. Four *Drosophila* species were trapped (*Drosophila melanogaster*, *Drosophila simulans*, *Drosophila subobscura*, and *Drosophila suzukii*), but *D. simulans* (*n* = 3,598) and *D. suzukii* (*n* = 2,378) dominated the species composition in the traps. Therefore, we included only these two dominating species in the statistical analysis. We used a generalized linear mixed model with counts as dependent variable assuming Poisson distribution and applying log link function. Yeast treatment (no yeast, non‐killer yeast, killer yeast), *Drosophila* species, and sex of the flies were used as fixed factors. Trap identity was included as a random factor in the model. Trap identity was chosen as a random effect after testing for alternative random effect structures (see Table S2 and Figure S2). The goal of choosing the random effect was to remove as much of the variance as possible that was due to yeast strain, killer virus strain and physical location of the trap in the field. As each trap was baited with a single yeast × virus combination, this single random effect counts for as much of the ecological variation and genetic variation as we can achieve without a rigorous experiment designed to control (or study) ecological, and genetic effects. Therefore, we believe that by using trap identity as a random effect we present a fair test of the fixed effects, namely presence of Killer phenotype, and contrasting the two *Drosophila* species. All analyses were done with IBM SPSS Statistics 25.

## 3 RESULTS

**FIGURE 1:**
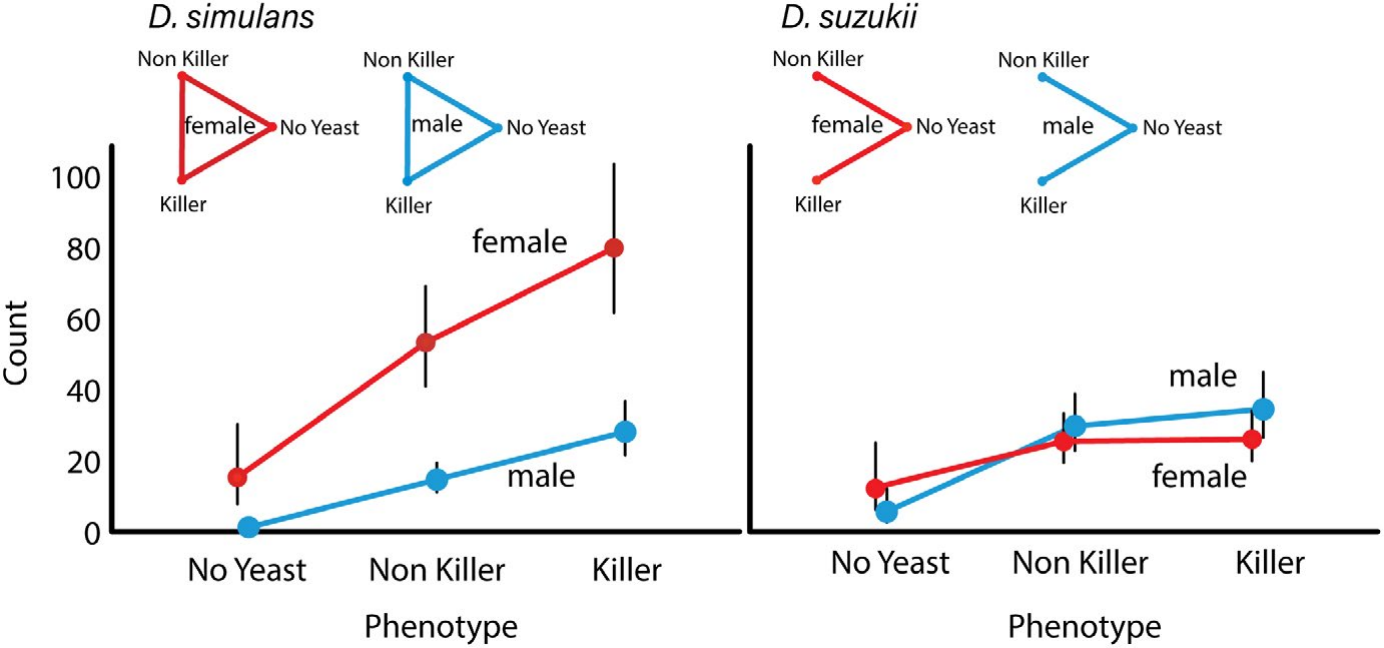
Results of a field experiment where traps containing no yeast (serve as control) and yeast without or with killer phenotype were placed for 72 hr in the vineyard. Panels show overall counts of attracted *Drosophila simulans* (left panel) and *Drosophila suzukii* (right panel) males (red) and females (blue). Symbols show generalized linear mixed model (see methods) estimated means and ∓1SE. Note that standard errors are asymmetric, because they are back transformed from the model that uses log link function. Triangle plots show results of pairwise comparisons of treatments for female and male *D. simulans* and *D. suzukii*. Treatments connected by line are statistically significant (*p* < 0.05) after adjusting for multiple testing. Pairwise testing was conducted using pairwise contrast option in generalized linear mixed model application available in SPSS 25

We found a significant three‐way interaction between yeast treatment, *Drosophila* species and fly sex (*F* = 7.69, *df*1 = 4, *df*2 = 144, *p* < 0.001, Figure 1, Table 1). Both *D. simulans* and *D. suzukii* were more attracted by *S. cerevisiae* compared with plain grape juice (Figure 1). *Drosophila suzukii* did not show increased attraction toward killer yeasts (Figure 1). In Europe, *D. suzukii* is an invasive species, laying its eggs in ripening fruits, while other *Drosophila* species prefer rotting fruits (Atallah et al., 2014). Here, we will focus discussion on results for *D. simulans*, which, contrary to *D. suzukii*, has previously been shown to be associated with *S. cerevisiae* in vineyards (Buser et al., 2014). *Saccharomyces cerevisiae* has been found in the gut and on the surface of wild *Drosophila* (Buser et al., 2014; Chandler et al., 2012). In *D. simulans,* both males and females were more attracted to killer yeast than non‐killer yeast (Figure 1). The pattern of attraction to yeast was much more pronounced in female *D. simulans* than in males (Figure 1).

**TABLE 1:** Results of generalized linear model with counts as dependent variable assuming Poisson distribution and applying log link function

Fixed effects						
Source	*F*	*df*1	*df*2	Sig.		
Corrected Model	125.860	11	144	0.000		
Yeast treatment	12.729	2	41	0.000		
*Drosophila* species	0.343	1	144	0.559		
*Drosophila* sex	74.152	1	144	0.000		
Species * yeast treatment	33.360	2	144	0.000		
Species * sex	55.012	1	144	0.000		
Species * sex * yeast treatment	7.694	4	144	0.000		

Random effect covariance	Estimate	*SE*	*Z*	Sig.	Lower 95% CI	Upper 95% CI
Trap number	0.280	0.070	4.030	0.000	0.172	0.456

## 4 DISCUSSION

We found that *D. simulans* were most attracted by the grape juice inoculated with yeast hosting the M satellite dsRNAs and the corresponding L‐A dsRNA helper virus. We are confident that this enhanced attraction is due to the killer phenotype, as we corrected for ecological and genetic variance as well. We also know from literature that neither killer phenotype nor yeast attraction seems to correlate with the taxonomic position (Arguello et al., 2013; Becher et al., 2018; Buser et al., 2014; Pieczynska et al., 2013). How the viruses contribute to attraction of the yeast or even manipulate their host to be more attractive requires further investigation. As increased attraction can be a win–win situation for both the yeast and the virus strain, disentangling whether effects are general (all yeast strains infected by the same virus strain induce attractiveness) or specific (level of attractiveness depends on yeast–virus strain combination) requires detailed and complex experiments. Here, results of our field experiment encourage us to discuss how viruses could be manipulating yeast host attractivity and how general this discovery could be.

*Assuming increased attraction is active host manipulation, what is the benefit to viruses?* The viruses benefit from dispersing to the new habitat patches with their yeasts, which is important for population growth and persistence in the temporary habitat mosaic of rotting fruit. But viruses also have additional interests in host dispersal. Viruses depend on *S. cerevisiae* engaging in sexual reproduction for transmission to uninfected yeast strains. Viruses transmit to new host genotypes when germinated yeast spores fuse, for example in the gut of insects (reviewed in Meriggi et al., 2020; Reuter et al., 2007; Stefanini et al., 2012, 2016). Unlike the vegetative yeast cells, the sexual yeast spores survive passage through the gut of insects (Reuter et al., 2007). Therefore, one alternative hypothesis for explaining killer yeast strains being more attractive to *Drosophila* could be that attractive yeast strains (independent of killer status) benefit from higher recombination when passaging the *Drosophila* gut. Chances to mate with a killer yeast spore are hence higher for attractive yeast, as passage through the insect gut is known to increase the likelihood of outbreeding in *S. cerevisiae* (Reuter et al., 2007; Stefanini et al., 2012, 2016). It would be informative to test this hypothesis by conducting attraction experiments with the same yeast genotypes that only differ concerning killer phenotype. This could be achieved by curing yeast cells from viruses (Fink & Styles, 1972; Wickner, 1974) and/or transfection of viruses into uninfected hosts (Pieczynska et al., 2017).

*What mechanisms are behind host manipulation to attract vectors?* Earlier studies have revealed substantial genetic diversity within *S. cerevisiae* (Gayevskiy & Goddard, 2012; Knight & Goddard, 2015; Peter et al., 2018) and connectivity among populations (Hyma & Fay, 2013; Knight & Goddard, 2015). *Drosophila* may be central in connecting the yeast populations (Goddard et al., 2010). Yeast volatiles have been shown to be involved in insect attraction and repulsion (summarized in Table 1, Gunther & Goddard, 2019). Volatiles have hence been suggested to be important components promoting mutualism between yeast and *Drosophila* (Buser et al., 2014; Christiaens et al., 2014). *Drosophila* locates and evaluates food source and quality based on olfactory cues. Yeast volatiles, not fruit volatiles, mediate *Drosophila* fitness by promoting adult attraction, oviposition, and larval development (Becher et al., 2012). Both yeasts and flowers share volatile signals that are attractive to *Drosophila* (Becher et al., 2018) and to which the flies respond via olfactory sensory neurons (Knaden et al., 2012).

A possible route for viruses to manipulate attraction would be through alteration of volatile composition released by the yeasts. Ferments with high killer activity differ for example in fermentation speed and volatile acidity (Maqueda et al., 2012). Acetic acid, one of the volatiles responsible for higher volatile acidity and produced by *S. cerevisiae* during fermentation, attracts *D. melanogaster* (Knaden et al., 2012). Although it is during this fermentation process when volatiles to attract *Drosophila* vectors are produced, the exact mechanism through which the toxin could interfere with volatile production remains speculation and needs further investigation.

In general, it is not uncommon that parasites manipulate chemosensory traits to increase transmission through insect‐vectored pathogens, as insects use volatiles to locate their host. For example, host plants infected with viruses are more attractive to insect vectors due to elevated volatile emission (Mauck et al., 2010) or through differences in volatile composition (Eigenbrode et al., 2002). Changes in the smell of infected hosts leading to higher attraction of the mosquito vector have for example been shown for hosts infected with malaria pathogens (De Moraes et al., 2014) and *Leishmania* (O'Shea et al., 2002). Fungal pathogens have been shown to induce attraction of its insect vector through the upregulation of volatiles of the host trees (McLeod et al., 2005), or through inducing mimicry of typical floral odors of host plants (Raguso & Roy, 1998). All these examples demonstrate the plausibility of higher vector attraction due to manipulation of volatile composition and/or emission level in the killer yeast system.

## 5 OUTLOOK

Viruses can disperse in two different ways depending on the mode of reproduction of *S. cerevisiae*. Both transmission routes are promoted due to a close association with insects. First, viruses can spread within the yeast genotype they inhabit through yeast dispersal when the vegetative cells are attached to the vector's body (Christiaens et al., 2014). Viruses thus disperse as their host genotype is dispersing. This transmission route can be studied in detail by mapping the distribution and colonization dynamics of particular yeast genotypes. With respect to killer phenotype, the interesting question here is whether higher attraction to vectors allows killer yeasts to spread faster and wider than non‐killer strains. Second, viruses disperse within yeast spores, which survive passage through insect guts and are very frequent in insect feces (Reuter et al., 2007). This dispersal mode through sexual reproduction of yeast enhances virus transmission into new host genotypes because of higher outcrossing possibility. Indeed, Reuter et al. (2007) suggest that yeast spores, and not vegetative cells, are the primary dispersal stage for *S. cerevisiae* species. This invites the possibility that viruses trigger sexual reproduction in their host yeast. Sporulation efficiency varies across different *S. cerevisiae* isolates (Gerke et al., 2006). Induced sexual spore production in the host is an interesting study question for future studies. If virus induces host sex, then the frequency of spore production should be higher in killer yeasts when probability of transmission by the vector is high.

One of the great research challenges in this context is that the importance of virus dispersal through vegetative yeast cells as well as virus transmission into new host genotypes by sexual reproduction still needs to be shown in natural populations. Under suitable environmental conditions, the virus–yeast interaction selects for monoclonal yeast populations in one local patch (low alpha diversity). At the same time, if killer yeasts are sexually active, they can spread the virus to uninfected yeast strains (increasing beta diversity). Effectively, this creates scenarios where monoclonal killer yeast populations maintain yeast diversity at the metapopulation level. Testing this hypothesis requires careful field surveys documenting both alpha and beta diversity of yeast metapopulations with and without viruses underlying the killer phenotype.

## CONFLICT OF INTEREST

The authors have no conflict of interest to declare.

## AUTHOR CONTRIBUTIONS

**Claudia C. Buser:** Conceptualization (lead); Data curation (lead); Formal analysis (equal); Investigation (lead); Methodology (lead); Project administration (lead); Supervision (lead); Writing‐original draft (lead); Writing‐review & editing (equal). **Jukka Jokela:** Conceptualization (supporting); Formal analysis (equal); Funding acquisition (equal); Methodology (supporting); Resources (equal); Writing‐original draft (supporting); Writing‐review & editing (equal). **Oliver Y. Martin:** Conceptualization (equal); Funding acquisition (equal); Investigation (supporting); Methodology (supporting); Supervision (equal); Writing‐original draft (equal); Writing‐review & editing (equal).

## Supporting information

Supplementary Material

## Data Availability

The Data used in the analysis of this paper can be found in the Dryad Data Repository (https://doi.org/10.5061/dryad.sn02v6x3r).
