# Supplementary material for: Scent of a killer: How could killer yeast boost its dispersal?
Source: Ecol Evol. 2021 May 1;11(11):5809–14. doi: 10.1002/ece3.7534 (PMC8207343; doi:10.1002/ece3.7534)
Supplement: Supplementary file 1 — Supplementary Material [file ECE3-11-5809-s001.docx]

# Supplementary Information

## Supplementary Tables:

Table S1: Information on the yeast strains used in the experiment.

| Isolate Name | Tree Order | Isolation | Geograpical origin | Clade | Collection/Providor |
| --- | --- | --- | --- | --- | --- |
| UC1_1v | 142 | Wine | France | 1. Wine/Eruopean | Krugylyak Laboratory |
| I14_1b | 390 | Wine | Italy | M1- Mosaic region 1 | Krugylyak Laboratory |
| NCYC_2743 | 296 | Wine | Spain | 1. Wine/Eruopean | National Collection of Yeast Cultures |
| Y12_1b | 223 | Palm wine | Ivory Coast | 26. Asian fermentation | Krugylyak Laboratory |
| YJM454_1b | 712 | Human, clinical | NA | M3- Mosaic region 3 | Krugylyak Laboratory |
| CLIB294_1b | 86 | Distillery | France | 1. Wine/Eruopean | CLIB collection |

Table S2: Results from testing for alternative random effect structures. Best model based on Akaike information criterion (AIC) implemented using trap identity as random effect. With trap identity as random effect as much of the variance as possible due to yeast/virus genetic and ecology was removed.

| Model | Random effects | AIC | LL-ratio | df | deviance | df | value/df | random effects | v(comp) |
| --- | --- | --- | --- | --- | --- | --- | --- | --- | --- |
| Count BY yeast treatment**Drosophila* species**Drosophila* sex | No random effects | 2642.255 | 1977.588 | 11 | 182.443 | 144 | 12.67 | 0 |  |
| Count BY yeast treatment**Drosophila* species**Drosophila* sex | Killer phenotype (KP) | 1205.593 |  |  |  |  |  | 4 | 0.261 |
| Count BY yeast treatment**Drosophila* species**Drosophila* sex | Yeast x KP, yeast, KP | 1020.933 |  |  |  |  |  | 32 | 0.047 |
| Count BY yeast treatment**Drosophila* species**Drosophila* sex | Yeast, KP | 1018.847 |  |  |  |  |  | 18 | 0.109 |
| Count BY yeast treatment**Drosophila* species**Drosophila* sex | Yeast | 1017.545 |  |  |  |  |  | 14 | 0.135 |
| Count BY yeast treatment**Drosophila* species**Drosophila* sex | Yeast x KP | 1017.545 |  |  |  |  |  | 14 | 0.135 |
| Count BY yeast treatment**Drosophila* species**Drosophila* sex | Trap number | 478.234 |  |  |  |  |  | 39 | 0.28 |

## Supplementary Figures:


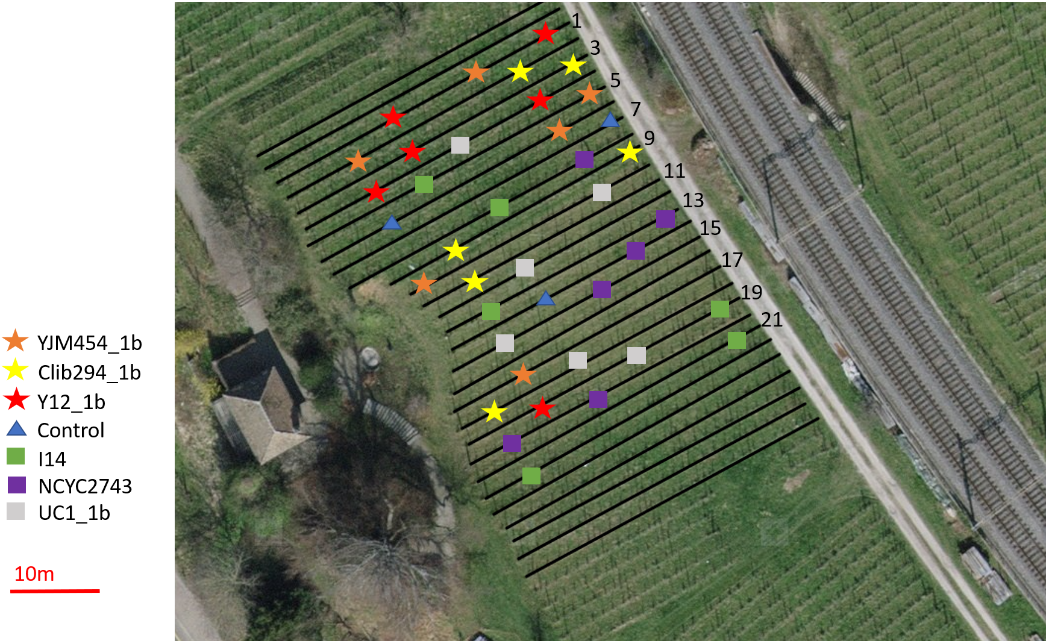


Figure S1: Distribution of traps at the sampling site. 36 traps were randomly placed in a vineyard (Schipf: 47.291925, 8.601796). Killer-yeasts are indicated with a star, non-killer yeasts with a square. Three traps containing plain grape juice served as controls (blue triangles).


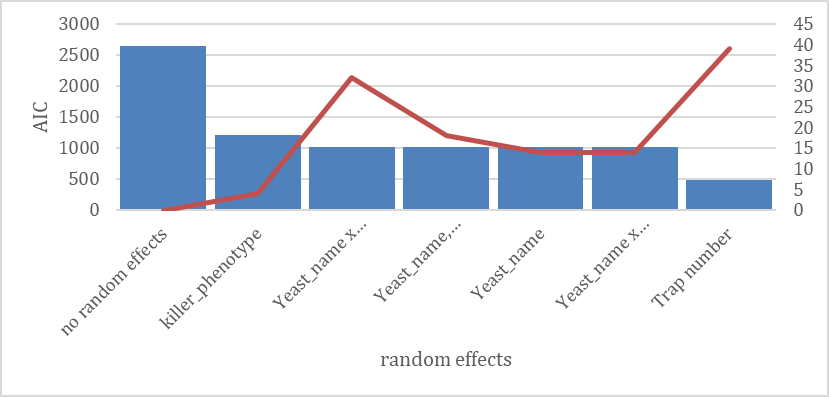


Figure S2: Akaike information criterion (AIC, left vertical axis) of generalized linear mixed models with different random effects. The model always included fixed effects of yeast treatment (no yeast, non-killer yeast, killer yeast), *Drosophila* species and sex and their interactions. Count of flies in the trap was used as a response variable assuming Poisson distribution and applying Log link function. The red line indicates the number of random effects in the model (right vertical axis).
